# Effectiveness and safety of ravulizumab for Japanese patients with atypical hemolytic uremic syndrome switched from eculizumab: an analysis of a post-marketing surveillance

**DOI:** 10.1007/s10157-025-02689-6

**Published:** 2025-06-14

**Authors:** Shuichi Ito, Hiroshi Hataya, Masanori Matsumoto, Akihiko Shimono, Hirofumi Teranishi, Masaki Okuda, Yoshitaka Miyakawa, Shoichi Maruyama

**Affiliations:** 1 Department of Pediatrics, Graduate School of Medicine, Yokohama City University, 3-9 Fukuura, Kanazawa-ku, Yokohama, Kanagawa 236-0004 Japan; 2 Department of General Pediatrics, Department of Nephrology, Tokyo Metropolitan Children’s Medical Center, 2-8-29 Musashidai, Fuchu, Tokyo 183-8561 Japan; 3 Department of Hematology and Blood Transfusion Medicine, Nara Medical University, 840 Shijo-cho, Kashihara, Nara 634-8522 Japan; 4 Alexion Pharma GK, 3-1-1 Shibaura, Minato-ku, Tokyo 108-0023 Japan; 5 Department of Hematology, Saitama Medical University, 38 Moroyama, Iruma-gun, Saitama 350-0495 Japan; 6 Department of Nephrology, Nagoya University Graduate School of Medicine, 65 Tsurumai-cho, Nagoya, Aichi 466-8550 Japan

**Keywords:** Atypical hemolytic uremic syndrome, Japan, Post-marketing surveillance, Ravulizumab, Safety, Thrombotic microangiopathy

## Abstract

**Background:**

Ravulizumab, a long-acting anti-C5 antibody, was approved for atypical hemolytic uremic syndrome (aHUS) in September 2020 in Japan. Post-marketing surveillance was mandated by local regulatory authorities to evaluate the effectiveness and safety of ravulizumab in patients with aHUS in real-world clinical practice.

**Methods:**

Patients with aHUS who switched from eculizumab to ravulizumab and received at least one dose of ravulizumab between September 2020 and December 2021 were enrolled. The effectiveness was evaluated by thrombotic microangiopathy (TMA) event-free status, defined as no sign of TMA recurrence and no initiation of plasma therapy/dialysis during ravulizumab treatment. The safety of ravulizumab was evaluated by summarizing the incidence of adverse events (AEs) and serious AEs.

**Results:**

This study included 33 patients (19 children and 14 adults). The median (range) duration of eculizumab treatment before the switch was 1233 (113–3240) days, and the duration of ravulizumab treatment was 351 (127–365) days. During ravulizumab treatment, TMA event-free status was achieved in 97.0% (32/33) of patients. The platelet count, lactate dehydrogenase levels, and serum creatinine levels remained stable during ravulizumab treatment. Twenty-nine AEs were reported in 13 patients, including nine serious AEs in seven patients. No meningococcal infections or deaths occurred during ravulizumab treatment. One patient discontinued treatment and died 478 days later from an unknown cause.

**Conclusions:**

This study confirmed the effectiveness and safety of ravulizumab in Japanese patients with aHUS after switching from eculizumab in a real-world setting.

**Supplementary Information:**

The online version contains supplementary material available at 10.1007/s10157-025-02689-6.

## Introduction

Atypical hemolytic uremic syndrome (aHUS) is a type of thrombotic microangiopathy (TMA) that is characterized by thrombocytopenia, hemolytic anemia, and organ damage, such as acute kidney injury [1, 2]. aHUS is defined as complement-mediated TMA, in which dysregulation of the complement pathway (caused by a combination of genetic predispositions or anti-complement factor H (CFH) antibodies and triggers/underlying conditions) mediates the onset of TMA [3, 4]. Variants in complement-related genes are detected in approximately 40%–60% of patients with aHUS [5]. However, aHUS is clinically diagnosed after other forms of TMA are excluded, such as thrombotic thrombocytopenic purpura, Shiga toxin-producing *Escherichia coli*-associated hemolytic uremic syndrome, and non-complement-mediated secondary TMA [4].

Two drugs, eculizumab and ravulizumab, are currently approved for aHUS in Japan. Eculizumab is a humanized monoclonal antibody against the complement protein C5 that has been approved in Japan for aHUS since 2013 [6]. The efficacy and safety of eculizumab have been reported in several clinical trials and real-world studies [7–15]. Post-marketing surveillance (PMS) studies have demonstrated the real-world effectiveness and safety of eculizumab among Japanese patients with aHUS [12–15]. Ravulizumab is a next-generation terminal complement inhibitor designed via targeted modification of eculizumab to achieve immediate and sustained terminal complement inhibition and an extended half-life and was approved for the treatment of aHUS in 2020 in Japan. The dosing regimen of ravulizumab is based on the patient’s body weight, and its dosing interval is longer (every 4 weeks for body weight ≥ 5 to < 40 kg, and every 8 weeks for body weight ≥ 40 kg) than that of eculizumab (every 2–3 weeks, depending on the patient’s age and body weight) [16].

Two multicenter, single-arm, 26-week, phase 3 studies have been conducted to evaluate the efficacy and safety of ravulizumab in adult (Study 311) and pediatric (Study 312) patients with aHUS [17–20]. Study 311 included adult, complement inhibitor-naïve patients (≥ 18 years of age; body weight ≥ 40 kg) [17], and Study 312 included pediatric patients (< 18 years of age; body weight ≥ 5 kg) who were complement inhibitor-naïve [18] and those who were previously treated with eculizumab [19]. Those clinical trial data showed that ravulizumab provided immediate, complete, and sustained C5 inhibition in patients with aHUS. Although 10 pediatric patients who were switched from eculizumab were enrolled in Study 312 [19], no phase 3 study was conducted in adult patients previously treated with eculizumab. Currently, evidence regarding the efficacy and safety of ravulizumab after switching from eculizumab has accumulated, as reported by the Global aHUS Registry and a German cohort study [21, 22].

Previous reports have described risk factors of TMA recurrence (e.g., presence of genetic variants, history of kidney transplantation, age of onset, and family history of aHUS) after treatment discontinuation [7, 23]. In patients with such risk factors, long-term treatment with anti-C5 antibody can be considered, and switching from eculizumab to ravulizumab may be preferred by patients [24]. This analysis using PMS data describes the characteristics of patients with aHUS who switched from eculizumab and evaluates the effectiveness and safety of ravulizumab in real-world clinical practice in Japan.

## Materials and methods

### Study design

PMS of ravulizumab was mandated by the Ministry of Health, Labour and Welfare of Japan. The PMS of ravulizumab enrolled patients with aHUS who were administered at least one dose of ravulizumab from September 2020 to December 2021 in Japan. The data cutoff for this analysis was December 2023. The observation period in patients who continued the treatment was 12 months from the start of ravulizumab treatment. Data on patient characteristics, clinical courses, and outcomes were collected using case report forms.

### Patients

Japanese patients with aHUS who received at least one dose of ravulizumab during the enrollment period and were switched from eculizumab to ravulizumab were included in this analysis. Complement inhibitor-naïve patients were not included in this analysis. The clinical diagnosis of aHUS was made by the attending physicians following the latest clinical guideline at the time [25–27].

### Treatment

Ravulizumab was administered by intravenous infusion according to the approved dosing for the indication [28]. Taking the patient’s body weight into account, the starting dose was 600–3000 mg as a single dose, followed by 300–3600 mg as a single dose 2 weeks after the first dose, and 300–3600 mg once every 4 or 8 weeks thereafter.

### Outcomes

The effectiveness endpoints were TMA event-free status, changes in hematologic and renal parameters including platelet (PLT) count, lactate dehydrogenase (LDH), hemoglobin and estimated glomerular filtration rate (eGFR), and dialysis status. TMA event-free status was defined as no decrease in PLT count > 25%, no decrease in PLT count to < 150 × 10^9^/L, no plasma exchange or infusion, and no initiation of dialysis, which was assessed at Weeks 14 and 26 and at Month 12 from the start of ravulizumab administration.

In the Japanese pediatric group, eGFR was calculated as follows: 110.2 × (reference serum creatinine level [sCr]/patient’s sCr) + 2.93. The reference sCr levels were calculated using the following equation, where X is body height: for male patients, − 1.259X^5^ + 7.815X^4^ − 18.57X^3^ + 21.39X^2^ − 11.71X + 2.628; for female patients, − 4.536X^5^ + 27.16X^4^ − 63.47X^3^ + 72.43X^2^ − 40.06X + 8.778 [29]. In the adult group, eGFR was calculated as follows [30]: for male patients, 194 × sCr (mg/dL)^−1.094^ × age (years)^−0.287^; for female patients, (194 × sCr (mg/dL)^−1.094^ × age (years)^−0.287^) × 0.739.

The safety endpoints during ravulizumab treatment were the incidence of adverse events (AEs), serious AEs, adverse drug reactions (ADRs), and serious ADRs. ADRs were defined as any AEs that were judged to be related to ravulizumab by the attending physician and were classified by the Medical Dictionary for Regulatory Activities System Organ Class and Preferred Term, version 25.0.

### Statistical analysis

Descriptive statistics were used to summarize patients’ demographic and clinical characteristics, including median (range) for continuous data and n (%) for categorical data. Background characteristics and efficacy outcomes were summarized by age category (pediatric: < 18 years old; adults: ≥ 18 years old). Laboratory data (PLT count, LDH, hemoglobin, and eGFR) were summarized as median (range) and collected at the following timepoints: aHUS diagnosis, last administration of eculizumab, first administration of ravulizumab, Week 14, Week 26, and Month 12. The safety outcomes were also summarized by age category and frequencies and percentages. The statistical analyses were performed using SAS software, version 9.4 or later (SAS Institute Inc., Cary, NC, USA).

## Results

### Patients

**Fig. 1:**
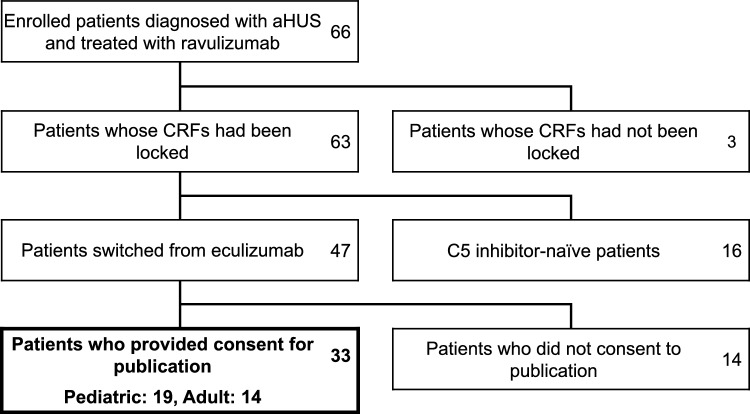
Patient disposition. This analysis included 33 patients (19 pediatric and 14 adult) who received ravulizumab treatment after switching from eculizumab and who provided consent for publication. *aHUS* atypical hemolytic uremic syndrome, *CRFs* case report forms

Among the 66 Japanese patients enrolled in the ravulizumab PMS, 33 patients who were switched from eculizumab and provided informed consent for publication were included in this analysis (Fig. 1). A total of 19 (57.6%) pediatric patients and 14 (42.4%) adult patients were included in this analysis (Table 1). Among them, 24.2% (8/33) of patients had a family history of aHUS and no patient had a previous diagnosis of aHUS or TMA before eculizumab treatment (Online Resource 1). At least one variant in complement genes was detected in 72.4% of patients (pediatric, 14; adults, 7; overall, 21) who received genetic testing (pediatric, 18; adults, 11; overall, 29) (Table 1). One variant of C3, two variants of factor H, and one variant of factor B were previously shown to be pathogenic (Online Resource 2). The proportion of patients who were positive for anti-CFH antibody was 37.5% (6) among all patients tested (16). Underlying complications at baseline were reported in four patients (21.1%) in the pediatric group and all 14 patients in the adult group (Table 1, Online Resource 3). As pretreatments for aHUS other than eculizumab, plasma therapy was reported in 68.4% (13/19) and 85.7% (12/14), dialysis was reported in 47.4% (9/19) and 92.9% (13/14), and kidney transplantation was reported in 0% (0/19) and 28.6% (4/14) of pediatric and adult patients, respectively (Table 1).

**Table 1:** Patient characteristics

Analysis item	Pediatric	Adult	Total
Number of patients	19 (57.6)	14 (42.4)	33 (100.0)
Age at first ravulizumab administration (years) (median, range)	8 (0, 17)	46 (22, 73)	14 (0, 73)
Sex, male	12 (63.2)	9 (64.3)	21 (63.6)
Family history of aHUS	5 (26.3)	3 (21.4)	8 (24.2)
Previous diagnosis of aHUS or TMA	0	0	0
Gene variant or anti-CFH antibody detected	14/18 (77.8)	9/11 (81.8)	23/29 (79.3)
Gene variant detected	14/18 (77.8)	7/11 (63.6)	21/29 (72.4)
Complement protein C3	5 (27.8)	3 (27.3)	8 (27.5)
Complement factor H	4 (22.2)	3 (27.3)	7 (24.1)
Complement factor I	0	0	0
Complement protein CD46 (membrane cofactor protein)	3 (16.7)	0 (0.0)	3 (10.3)
Complement factor B	1 (5.6)	1 (9.1)	2 (6.9)
Thrombomodulin	0	0	0
Diacylglycerol kinase epsilon	1 (5.6)	0 (0.0)	1 (3.4)
Other	1 (5.6)^a^	1 (9.1)^b^	2 (6.9)
Anti-CFH antibody detected	3/9 (33.3)	3/7 (42.9)	6/16 (37.5)
Medical history/complications	4 (21.1)	14 (100.0)	18 (54.5)
Cobalamin metabolism disorder	0	0	0
Autoimmune disease/collagen disease	0	2 (14.3)	2 (6.1)
Accelerated malignant hypertension	0	0	0
Malignant tumor	0	0	0
Infection	0	0	0
Pregnancy-related HELLP syndrome, eclampsia	0	0	0
Drug-induced TMA	0	0	0
Acute pancreatitis	0	0	0
Hematopoietic stem cells/TMA after organ transplantation	0	0	0
Other	4 (21.1)	14 (100.0)	18 (54.5)
Pretreatment for aHUS (before eculizumab treatment)			
Plasma therapy	13 (68.4)	12 (85.7)	25 (75.8)
Blood transfusion	9 (47.4)	10 (71.4)	19 (57.6)
Plasma exchange	12 (63.2)	11 (78.6)	23 (69.7)
Fresh frozen plasma infusion	5 (26.3)	3 (21.4)	8 (24.2)
Kidney transplant	0	4 (28.6)	4 (12.1)
Dialysis	9 (47.4)	13 (92.9)	22 (66.7)

Data are n (%) unless otherwise specified

*aHUS* atypical hemolytic uremic syndrome, *CFH* complement factor H, *CFHR* complement factor H-related, *HELLP* hemolysis, elevated liver enzymes, and low platelets, *TMA* thrombotic microangiopathy

^a^*CFHR1* and *CFHR3* deletion

^b^*CFH* and *CFHR* fusion (no details reported)

The median (range) duration of treatment with eculizumab before switching to ravulizumab was 1233 (113–3240) days (Table 2). In four pediatric patients, the number of days between the last dose of eculizumab and the first dose of ravulizumab was recorded as 784, 737, 815, and 808 days in the PMS. These four patients participated in the ravulizumab phase 3 trial (Study 312) [19]. Although they switched from eculizumab to ravulizumab without delay, PMS had no record of ravulizumab treatment during the period of the ravulizumab clinical trial (784, 737, 815, and 808 days) (Online Resource 1). The median (range) duration of ravulizumab treatment was 351 (127–365) days among all patients. In all cases, the dose interval of ravulizumab was longer than that of eculizumab (Online Resource 4). One adult patient with aHUS with anti-CFH antibody discontinued ravulizumab after subsequently testing negative for anti-CFH antibody and died 478 days later from an unknown cause. The death was judged not to be related to ravulizumab (Online Resource 5).

**Table 2:** Prior treatment with eculizumab and ravulizumab

Analysis item	Pediatric	Adult	Total
Median days of eculizumab treatment before switching to ravulizumab, days (median [range])	1466 (196, 3096)	1195.5 (113, 3240)	1233 (113, 3240)
Time from the last administration of eculizumab to the first administration of ravulizumab, days (median [range])	16 (12, 815)	15 (13, 17)	15 (12, 815)
Duration of ravulizumab treatment, days (median [range])	351 (337, 365)	351 (127, 365)	351 (127, 365)
Discontinuation of ravulizumab during observation period (n [%])	0	1 (7.1)	1 (3.0)

All patients were vaccinated against *Neisseria meningitidis*. One patient who had received prophylactic antibiotics during eculizumab treatment was vaccinated after ravulizumab initiation.

### Effectiveness

**Fig. 2:**
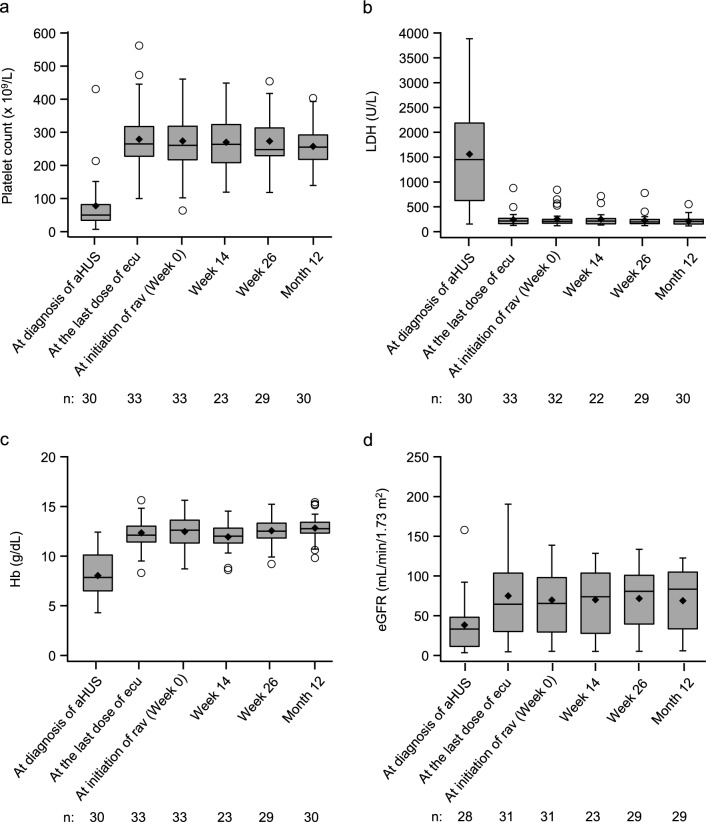
Laboratory data before and after ravulizumab treatment. Platelet count (**a**), lactate dehydrogenase (**b**), hemoglobin (**c**), and estimated glomerular filtration rate (**d**) are indicated by box plot at the diagnosis of aHUS, at the last dose of eculizumab (ecu) and during ravulizumab (rav) treatment, at initiation (Week 0), Week 14, Week 26, and Month 12. The number of patients at each observation point is indicated by n. Boxes represent the median and first (Q1) and third quartiles (Q3), whiskers represent the minimum and maximum (excluding outliers indicated by circles), and diamonds represent mean values. Outliers were defined as values outside the interval of Q1 − 1.5 (Q3 − Q1) and Q1 + 1.5 (Q3 − Q1). *aHUS* atypical hemolytic uremic syndrome*, eGFR* estimated glomerular filtration rate, *Hb* hemoglobin, *LDH* lactate dehydrogenase

The hematologic and renal parameters in patients remained stable during ravulizumab treatment (Fig. 2). Median PLT, hemoglobin, and eGFR values increased from the diagnosis of aHUS to the last dose of eculizumab and remained stable up to Month 12 after starting treatment with ravulizumab. The median value of LDH decreased from the diagnosis of aHUS to the last dose of eculizumab and remained stable up to Month 12 after starting treatment with ravulizumab.

TMA event-free status was met by 97.0% (32/33) of patients (Table 3). TMA event-free status was not met in one patient because the PLT count temporarily decreased by ≥ 25%; the PLT count was 365 × 10^9^/L at the start of administration of ravulizumab and decreased to 268 × 10^9^/L (26.7% decrease) at Week 14. Thereafter, the PLT count increased to 316 × 10^9^/L at Week 26 without any additional therapy. The attending physician did not report any recurrence of TMA during this period. Two patients on dialysis at ravulizumab initiation remained on dialysis throughout the observation period (Table 3).

**Table 3:** Effectiveness outcomes

Analysis item	Pediatric	Adult	Total
TMA event-free status	18/19 (94.7)	14/14 (100.0)	32/33 (97.0)
Reported recurrence of TMA	0	0	0
Dialysis at the initiation of ravulizumab	0	2/14 (14.3)	2/33 (6.1)
Dialysis withdrawal after ravulizumab administration	–	0/2 (0)	0/2 (0)
Death during ravulizumab treatment	0	0	0

Data are n (%)

*TMA* thrombotic microangiopathy

### Safety

AEs and serious AEs were reported in 39.4% (n = 13, 29 events) and 21.2% (n = 7, nine events) of patients, respectively (Table 4). The overall incidence of ADRs was 24.2% (n = 8, 16 events) and that of serious ADRs was 12.1% (n = 4, five events; including gastroenteritis *Salmonella* infection, upper respiratory tract infection, *Salmonella* bacteremia infection, pyrexia, and infusion-related reaction). No deaths were observed during ravulizumab treatment.

**Table 4:** Overview of adverse events

Analysis item	Pediatric		Adult		Total	
	(n = 19)		(n = 14)		(n = 33)	
	Patients, n (%)	Events	Patients, n (%)	Events	Patients, n (%)	Events
Adverse event	8 (42.1)	13	5 (35.7)	16	13 (39.4)	29
Serious adverse event	5 (26.3)	6^a^	2 (14.3)	3^b^	7 (21.2)	9
Adverse drug reaction	6 (31.6)	9	2 (14.3)	7	8 (24.2)	16
Serious adverse drug reaction	3 (15.8)	4	1 (7.1)	1	4 (12.1)	5

^a^Includes gastroenteritis *salmonella*, upper respiratory tract infection, *salmonella* bacteremia, vascular device infection, pyrexia, and infusion related reaction (one event each)

^b^Includes pyelonephritis acute, pyrexia, and shunt stenosis (one event each)

ADRs observed in ≥ 2 patients overall were upper respiratory tract infection (in two pediatric patients), pyrexia (in two adult patients), and infusion-related reaction (in two pediatric patients). Total complement activity (CH50) was reported to be elevated above normal in one patient, but no infection was reported in association with the CH50 elevation, which resolved after 15 days, and no recurrence of TMA was reported in this patient (Table 5).

**Table 5:** Adverse drug reactions by System Organ Class and Preferred Term

	Pediatric (n = 19)		Adult (n = 14)		Total (n = 33)	
Number of adverse events	13		16		29	
Person-years	9.5		7.0		16.5	
System Organ Class Preferred Term	**ADR**	**Serious ADR**	**ADR**	**Serious ADR**	**ADR**	**Serious ADR**
All	9 (0.95)	4 (0.42)	7 (1.00)	1 (0.14)	16 (0.97)	5 (0.30)
Infections and infestations^a^	4 (0.42)	3 (0.32)	0	0	4 (0.24)	3 (0.18)
Gastroenteritis *salmonella*	1 (0.11)	1 (0.11)	0	0	1 (0.06)	1 (0.06)
Upper respiratory tract infection	2 (0.21)	1 (0.11)	0	0	2 (0.12)	1 (0.06)
* Salmonella* bacteremia	1 (0.11)	1 (0.11)	0	0	1 (0.06)	1 (0.06)
Psychiatric disorders	0	0	1 (0.14)	0	1 (0.06)	0
Anxiety	0	0	1 (0.14)	0	1 (0.06)	0
Nervous system disorders	1 (0.11)	0	1 (0.14)	0	2 (0.12)	0
Headache	0	0	1 (0.14)	0	1 (0.06)	0
Lethargy	1 (0.11)	0	0	0	1 (0.06)	0
Vascular disorders	1 (0.11)	0	0	0	1 (0.06)	0
Vasculitis	1 (0.11)	0	0	0	1 (0.06)	0
Gastrointestinal disorders	0	0	2 (0.29)	0	2 (0.12)	0
Abdominal pain	0	0	1 (0.14)	0	1 (0.06)	0
Nausea	0	0	1 (0.14)	0	1 (0.06)	0
General disorders and administration site conditions	0	0	3 (0.43)	1 (0.14)	3 (0.18)	1 (0.06)
Malaise	0	0	1 (0.14)	0	1 (0.06)	0
Pyrexia	0	0	2 (0.29)	1 (0.14)	2 (0.12)	1 (0.06)
Investigations	1 (0.11)	0	0	0	1 (0.06)	0
Total complement activity increased	1^b^ (0.11)	0	0	0	1^a^ (0.06)	0
Injury, poisoning and procedural complications	2 (0.21)	1 (0.11)	0	0	2 (0.12)	1 (0.06)
Infusion related reaction	2 (0.21)	1 (0.11)	0	0	2 (0.12)	1 (0.06)

Data are n (events per person-years)

*ADR* adverse drug reaction

^a^There were no cases of meningococcal infection (an adverse event of special interest)

^b^Patient did not experience TMA recurrence

The most common ADR was infection-related (events/person-years: 0.24; Table 5), but no meningococcal infections were reported. The rate of meningococcal vaccination at the start of ravulizumab administration was 97.0% (32/33), and one unvaccinated patient received antibiotics.

## Discussion

We report the characteristics of 33 Japanese patients with aHUS who were switched from eculizumab to ravulizumab and enrolled in this PMS. This study provides the first real-world evidence of the effectiveness and safety of ravulizumab in Japan. A large proportion (97.0%, 32/33) of patients met TMA event-free status at Month 12 after switching to ravulizumab. The hematologic and renal parameters of the patients remained stable from the last dose of eculizumab until Month 12 after the start of ravulizumab treatment. The most common ADRs were related to infection (events/person-years: 0.24), but no meningococcal infections were observed.

All patients received eculizumab treatment for over 3 months (90 days) before switching to ravulizumab with the median (range) duration of eculizumab treatment being 1233 (113–3240) days. The duration of treatment with C5 inhibitors may depend on the patient’s risk of TMA recurrence and must be individualized [31]; the duration of treatment with a C5 inhibitor may be longer in patients at high risk.

Risk factors for TMA include genetic variants, family history of aHUS, history of kidney transplantation, and history of dialysis [7, 23, 32–34]. Patients with these factors may have a high risk of TMA recurrence and subsequent irreversible organ damage and accumulated renal impairment after discontinuation of treatment with C5 inhibitors [33]. In this study, genetic variants were found in 72.4% (21/29) of patients, and the presence of such variants was reported to be a risk factor for TMA recurrence in aHUS [32, 33]. A history of kidney transplantation was reported in 28.6% (4/14) of adult patients in this study. In terms of other risk factors, in the present study, the proportions of patients with a family history of aHUS and a history of dialysis were 24.2% (8/33) and 66.7% (22/33), respectively. Notably, 30 of the 33 patients (90.9%) had at least one of the abovementioned four risk factors, and 29 of these 30 patients continued treatment with ravulizumab during the observation period. A recent patient preference study comparing eculizumab and ravulizumab showed that the surveyed patients and caregivers had an overall preference for ravulizumab over eculizumab for the treatment of aHUS, primarily due to reduced infusion frequency [24].

Effectiveness endpoints in our study included TMA event-free status, which was modified from the criteria in an eculizumab clinical trial to include no decrease in PLT count to < 150 × 10^9^/L [8]. The original TMA event-free status was defined as no decrease in PLT count of ≥ 25%, no plasma exchange or infusion, and no initiation of dialysis. In the patients who switched from eculizumab to ravulizumab, PLT count was normalized to ≥ 150 × 10^9^/L before ravulizumab initiation. Because a decrease in PLT count is a sign of TMA recurrence, we included no decrease in PLT count to < 150 × 10^9^/L as part of the TMA event-free status effectiveness endpoint in our study. One pediatric patient did not meet TMA event-free status because of a decrease in PLT count of ≥ 25%. However, the PLT count remained in the normal range in this patient, who recovered without additional therapy. Based on this observation, the attending physician did not consider this event as TMA recurrence.

In this analysis, among 22 patients who had received dialysis before eculizumab treatment, 20 patients (90.9%) had already withdrawn from dialysis before switching to ravulizumab, and two patients (9.1%) remained on dialysis; neither of them could withdraw from dialysis during ravulizumab treatment. Because they had complications (one of these patients had chronic kidney disease with type 2 diabetes mellitus, and the other had chronic kidney disease with hyperuricemia) and their duration of dialysis during eculizumab treatment was relatively long (1171 and 281 days, respectively), the progression of the patients’ reduced renal function may have been irreversible before switching to ravulizumab. Even in patients with irreversible kidney damage, the risk of extrarenal organ damage after a recurrence of TMA still exists; therefore, continuation or discontinuation of complement inhibitor treatment needs to be carefully considered.

In contrast to the large amount of safety data for eculizumab [7–15], few reports of real-world data for ravulizumab are available [35]. In our study, serious AEs were reported in 21.2% of patients treated with ravulizumab after switching from eculizumab, whereas a previous study reported serious AEs in 60.9% of patients treated with eculizumab [36, 37]. The lower frequency of serious AEs during ravulizumab treatment might reflect the stable condition in patients who had been treated with eculizumab before switching to ravulizumab. No unexpected serious ADRs were observed compared with data for eculizumab [12–15] or previous clinical trials on ravulizumab [15–17]. Increased CH50 above the normal level was reported as an ADR in one patient, but recurrence of TMA was not observed, and the CH50 level decreased later. Discordance between free C5 and CH50 levels under ravulizumab treatment has been reported [38, 39]; therefore, the CH50 assay may not be optimal to assess the effects of ravulizumab treatment.

This study has some limitations. In the PMS case report forms, sufficient clinical data before ravulizumab treatment (e.g., changes in laboratory parameters before/after eculizumab treatment, duration of pretreatment for aHUS, and patient quality of life) were not collected, which may hinder understanding of the patients’ clinical courses. The percentage of anti-CFH antibody-positive patients (pediatric, 33.3%; adult, 42.9%) in the present study was higher than that reported in previous PMS studies of eculizumab (pediatric, 18.2%; adult, 0%) [13, 14]. This study included patients who had been enrolled after completion of the eculizumab PMS; however, because the results of anti-CFH antibody assays can vary according to systems and no consensus cutoff value exists [40], the results should be carefully interpreted. For the safety analysis, the relationship between ADRs (e.g., *Salmonella* infection) and ravulizumab largely depended on reports from the attending physicians, and a direct causal relationship was not confirmed. The generalizability of the results is limited because the sample size was small, and this study included only Japanese patients.

## Conclusion

The effectiveness and safety of ravulizumab were demonstrated in Japanese patients with aHUS switched from eculizumab in real-world clinical practice. Furthermore, hematologic and renal parameters remained stable in the transition from eculizumab to ravulizumab. Compared with eculizumab, treatment with ravulizumab, a long-acting C5 inhibitor, could reduce the treatment burden on patients with aHUS and their caregivers.

## Supplementary Information

Below is the link to the electronic supplementary material.

Supplementary file1 (DOCX 93 KB)
