# Supplementary material for: Effectiveness and safety of ravulizumab for Japanese patients with atypical hemolytic uremic syndrome switched from eculizumab: an analysis of a post-marketing surveillance
Source: Clin Exp Nephrol. 2025 Jun 14;29(11):1608–17. doi: 10.1007/s10157-025-02689-6 (PMC12568904; doi:10.1007/s10157-025-02689-6)
Supplement: Supplementary file 1 — Supplementary file1 (DOCX 93 KB) [file 10157_2025_2689_MOESM1_ESM.docx]

# **Supplementary materials**

**Effectiveness and safety of ravulizumab for Japanese patients with atypical hemolytic uremic syndrome switched from eculizumab: an analysis of a post-marketing surveillance**

| **Content** | **Page** |
| --- | --- |
| **Online Resource 1.** Characteristics of the individual patients | 2, 3 |
| **Online Resource 2.** Gene variants and allele frequency in the gnomAD database | 4 |
| **Online Resource 3.** Underlying complications | 5 |
| **Online Resource 4.** Timing of ravulizumab infusion | 6 |
| **Online Resource 5.** Details of the patient who discontinued ravulizumab | 7 |

**Online Resource 1.** **Characteristics of the individual patients**

| **Patient No.** | **Pediatric/Adult (Age)** | **Sex** | **Family history** | **Previous diagnosis of aHUS** | **Genetic variant** | **History of kidney transplant** | **History of dialysis before ravulizumab** | **On dialysis before ravulizumab initiation** | **Days of eculizumab treatment before switching to ravulizumab** | **Time from the last administration of eculizumab to the first administration of ravulizumab** | **Days of ravulizumab treatment** |
| --- | --- | --- | --- | --- | --- | --- | --- | --- | --- | --- | --- |
| 1 | Pediatric (11) | Male |  |  | Negative |  |  |  | 1996 | 15 | 365 |
| 2 | Pediatric (7) | Female |  |  | NA |  |  |  | 2731 | 22 | 350 |
| 3 | Pediatric (11) | Male |  |  | Positive  (MCP) |  | Yes |  | 2928 | 16 | 351 |
| 4 | Pediatric (5) | Female | Yes |  | Positive  (CFH) |  | Yes |  | 1987 | 16 | 351 |
| 5 | Pediatric (0) | Female | Yes |  | Positive  (CFH) |  |  |  | 196 | 22 | 351 |
| 6^a^ | Pediatric (5) | Male | Yes |  | Positive  (CFH) |  | Yes |  | 1064 | 784 | 337 |
| 7^a^ | Pediatric (3) | Male | Yes |  | Negative |  |  |  | 234 | 737 | 365 |
| 8^a^ | Pediatric (11) | Male | Yes |  | Positive  (CFB) |  | Yes |  | 1702 | 815 | 337 |
| 9^a^ | Pediatric (14) | Male |  |  | Positive  (MCP) |  | Yes |  | 1511 | 808 | 337 |
| 10 | Pediatric (1) | Female |  |  | Positive  (CFH) |  |  |  | 217 | 22 | 351 |
| 11 | Pediatric (3) | Female |  |  | Positive  (C3) |  | Yes |  | 251 | 15 | 350 |
| 12 | Pediatric (12) | Female |  |  | Positive  (C3) |  |  |  | 1188 | 16 | 365 |
| 13 | Pediatric (17) | Female |  |  | Negative |  |  |  | 1233 | 15 | 351 |
| 14 | Pediatric (14) | Male |  |  | Positive  (C3, MCP) |  |  |  | 2437 | 43 | 358 |
| 15 | Pediatric (10) | Male |  |  | Positive  (CFHR1 del, CFHR3 del) |  |  |  | 1677 | 36 | 358 |
| 16 | Pediatric (15) | Male |  |  | Positive  (C3) |  |  |  | 980 | 15 | 344 |
| 17 | Pediatric (8) | Male |  |  | Positive  (DGKε) |  | Yes |  | 3096 | 14 | 357 |
| 18 | Pediatric (3) | Male |  |  | Positive  (C3) |  | Yes |  | 846 | 15 | 351 |
| 19 | Pediatric (7) | Male |  |  | Negative |  | Yes |  | 1466 | 12 | 351 |
| 20 | Adult (40s) | Male |  |  | NA | Yes | Yes | Yes | 1171 | 15 | 351 |
| 21 | Adult (70s) | Female |  |  | NA |  | Yes |  | 180 | 15 | 365 |
| 22 | Adult (30s) | Female |  |  | Positive  (C3) |  |  |  | 185 | 13 | 365 |
| 23 | Adult (60s) | Male |  |  | NA |  | Yes |  | 2605 | 15 | 351 |
| 24 | Adult (30s) | Female |  |  | Positive  (C3, CFB) |  | Yes | Yes | 281 | 16 | 353 |
| 25 | Adult (70s) | Male | Yes |  | Positive  (CFH) |  | Yes |  | 1479 | 15 | 350 |
| 26 | Adult (40s) | Male |  |  | Negative |  | Yes |  | 113 | 15 | 127 (Discontinued) |
| 27 | Adult (20s) | Female |  |  | Negative |  | Yes |  | 118 | 15 | 349 |
| 28 | Adult (50s) | Female |  |  | Negative |  | Yes |  | 549 | 15 | 351 |
| 29 | Adult (50s) | Male |  |  | Positive  (CFH-CFHR fusion) |  | Yes |  | 1220 | 15 | 351 |
| 30 | Adult (40s) | Male | Yes |  | Positive  (C3) | Yes | Yes |  | 1388 | 16 | 351 |
| 31 | Adult (30s) | Male | Yes |  | Positive  (CFH) |  | Yes |  | 2080 | 15 | 351 |
| 32 | Adult (40s) | Male |  |  | Negative | Yes | Yes |  | 1743 | 15 | 351 |
| 33 | Adult (40s) | Male |  |  | Positive  (CFH) | Yes | Yes |  | 3240 | 17 | 365 |

^a^Patients who had been enrolled in the phase 3 clinical trial of ravulizumab before PMS registration were registered in the PMS after the clinical trial.

*aHUS* atypical hemolytic uremic syndrome, *CFB* complement factor B, *CFH* complement factor H, *CFHR* complement factor H-related, *DGKε* diacylglycerol kinase ε, *MCP* membrane cofactor protein, *NA* not available, *PMS* post-marketing surveillance.

**Online Resource 2.** **Gene variants and allele frequency in the gnomAD database**

| **Allele** | **gnomAD**  **(East Asian)** | **Note [reference]** |
| --- | --- | --- |
|  |  |  |
| C3 p.Ile1157Thr | None | Pathogenic [1] |
| C3 p.Met1604Thr | 0.0005268 |  |
| C3 p.Ser182Pro | None |  |
| C3 p.Arg1042Leu | None |  |
| C3 p.Lys1105Gln+ p.Trp1077Arg | None | Lys1105Gln [2] |
| Factor H p.Ser1191Trp | None | Pathogenic CFH variant [3] |
| Factor H p.Arg1215Gln | None | Pathogenic CFH variant [3] |
| Factor H p.Val62Ile | 0.4155 | Benign CFH variant [3] |
| Factor H p.His402Tyr | 0.9410 | Benign CFH variant [3] |
| Factor H exon21-2212CNV | None |  |
| Factor H p.Phe176Leu | 0.0003126 |  |
| Factor B p.Lys350Asn | None | Pathogenic [4] |
| Factor B p.Ile502Thr | 0.00002519 |  |

1. Matsumoto T, Fan X, Ishikawa E, et al. Analysis of patients with atypical hemolytic uremic syndrome treated at the Mie University Hospital: concentration of C3 p.I1157T mutation. Int J Hematol. 2014;100:437–42.

2. Yoshida Y, Miyata T, Matsumoto M, et al. A novel quantitative hemolytic assay coupled with restriction fragment length polymorphisms analysis enabled early diagnosis of atypical hemolytic uremic syndrome and identified unique predisposing mutations in Japan. PLOS One. 2015;10:e0124655.

3. Merinero HM, Zhang Y, Arjona E, et al. Functional characterization of 105 factor H variants associated with aHUS: lessons for variant classification. Blood. 2021;138:2185–201.

4. Funato M, Uemura O, Ushijima K, et al. A complement factor B mutation in a large kindred with atypical hemolytic uremic syndrome. J Clin Immunol. 2014;34:691–5.

*CFH* complement factor H.

**Online Resource 3.** **Underlying complications**

| **Pediatric (n = 4/19)** |
| --- |
| Hypertension |
| Cardiac failure, protein urine present |
| Cardiac failure congestive, congenital megacolon, renal dysplasia, renal transplant, short bowel syndrome, single functional kidney |
| Membranoproliferative glomerulonephritis, renal hypertension |
| **Adult (n = 14/14)** |
| Hyperkalemia, hyperphosphatemia, hypertension, chronic kidney disease, type 2 diabetes mellitus |
| Mitral valve incompetence, acute kidney injury |
| Hypertension, adjustment disorder |
| Depression, gastroesophageal reflux disease, hypertension, hyperuricemia, insomnia, chronic kidney disease |
| Cardiac failure acute, hypertension, hyperuricemia, left ventricular hypertrophy, nephrogenic anemia, chronic kidney disease, acute kidney injury |
| Constipation, Henoch–Schönlein purpura, hypertension, hyperuricemia |
| Diabetes mellitus, hypertension, hyperuricemia, dyslipidemia, diabetic nephropathy |
| Cardiac failure, dysmenorrhea, hypertension, chronic kidney disease |
| Collagen disorder, mitral valve incompetence, peripheral arterial occlusive disease, microscopic polyangiitis |
| Hypertension, hyperuricemia |
| Adrenal insufficiency, angina pectoris, extrasystoles, hypertension, hyperuricemia, neurogenic bladder, osteoporosis, renal transplant, dyslipidemia |
| Gastroesophageal reflux disease, hypertension, hyperuricemia |
| Hypertension |
| Chronic gastritis, hemorrhoids, hiatus hernia, renal hypertension, renal transplant, rhinitis allergic, nephrogenic anemia, chronic kidney disease |

**Online Resource 4.** **Timing of ravulizumab infusion**


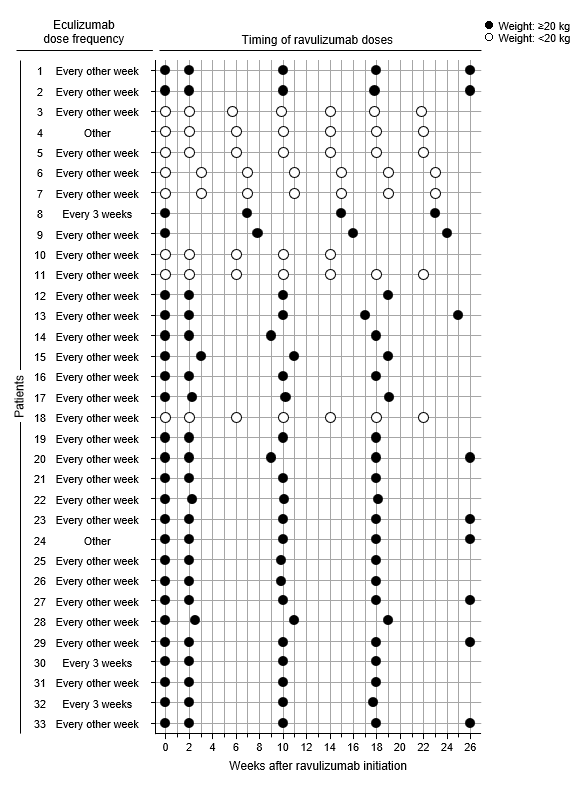


**Online Resource 5.** **Details of the patient who discontinued ravulizumab**

| **Patient background and clinical course** |
| --- |
| Man in his 40s with no family history of TMA, previous diagnosis of TMA or aHUS before eculizumab treatment, or genetic variant. Anti-CFH antibody was positive at the initiation of eculizumab treatment.  Patient was treated with eculizumab for 113 days and switched to ravulizumab. Ravulizumab treatment was stopped after 127 days because anti-CFH antibody became negative. Patient died at home 478 days later from an unknown cause. No relationship between ravulizumab and death was reported. |
| **Underlying complications at ravulizumab initiation** |
| Diabetes mellitus, hypertension, hyperuricemia, dyslipidemia, diabetic nephropathy |
| **Adverse events during ravulizumab treatment** |
| Lipids increased, dyslipidemia aggravated, hyperuricemia, hypertension |

*aHUS* atypical hemolytic uremic syndrome, *CFH* complement factor H, *TMA* thrombotic microangiopathy.
